# Clinical Features of Sarcomatoid Carcinoma (Carcinosarcoma) of the Urinary Bladder: Analysis of 221 Cases

**DOI:** 10.1155/2010/454792

**Published:** 2010-07-18

**Authors:** Jue Wang, Fen Wei Wang, Chad A. LaGrange, George P. Hemstreet III, Anne Kessinger

**Affiliations:** ^1^Section of Oncology-Hematology, Department of Internal Medicine, University of Nebraska Medical Center, Omaha, NE 68198-7680, USA; ^2^Department of Internal Medicine, Creighton University School of Medicine, Omaha, NE 68131, USA; ^3^Urologic Surgery Section, Department of Surgery, University of Nebraska Medical Center, Omaha, NE 68198-2360, USA

## Abstract

*Background*. Urinary bladder sarcomatoid carcinoma (carcinosarcoma) is rare. The objective of this study was to examine the epidemiology, natural history, and prognostic factors of urinary bladder carcinosarcoma using population-based registry. *Methods*. The Surveillance, Epidemiology, and End Results (SEER) Program database was used to identify cases by tumor site and histology codes. The association between clinical and demographic characteristics and long-term survival was examined. *Results*. A total of 221 histology confirmed cases were identified between 1973 and 2004, this accounted for approximately 0.11% of all primary bladder tumors during the study period. Median age of the patients was 75 years (range 41–96). Of the patients with a known tumor stage (*N* = 204), 72.5% had a regional or distant stage; 98.4% of patients with known histology grade (*N* = 127), had poorly or undifferentiated histology. Multiple primary tumors were indentified in about 40% of study subjects. The majority of patients (95.9%) received cancer directed surgery, 35.8% had radical or partial cystectomy, 15.8% of patients received radiation therapy combination with surgery. The median overall survival was 14 months (95% CI 7–21 months). 1-, 5-, and 10-year cancer specific survival rate were 53.9%, 28.4% and 25.8%. In a multivariate analysis, only tumor stage was found to be a significant prognostic factor for disease-specific survival. *Conclusions*. Urinary bladder carcinosarcoma commonly presented as high grade, advanced stage and aggressive behavior with a poor prognosis. Emphasis on early detection, including identification of risk factors is needed to improve the outcome for patients with this malignancy.

## 1. Introduction

Sarcomatoid carcinoma (Carcinosarcomas) is defined by the World Health Organization as a biphasic tumor consisting of malignant epithelial and mesenchymal elements [1]. Carcinosarcoma of the urinary bladder is a rare neoplasm. Approximately 70 cases have been reported in the literature, most often as a single case report or limited series [2–10] with focus on histopathological characteristics. Microscopically, carcinosarcomas are biphasic tumors made of an intimate admixture of carcinomatous and sarcomatous components with abrupt or gradual transition from one to the other. In most cases, the epithelial component consists of high-grade transitional cell carcinoma with possible epidermoid and/or glandular differentiation, while the heterologous component consists of chondrosarcoma, malignant fibrous histiocytoma, osteosarcoma, leiomyosarcoma, fibrosarcoma, or rhabdomyosarcoma [7–10]. Clinically, carcinosarcomas occur more commonly in older males, and present as advanced stage, rapidly growing polypoid neoplasms [1–6]. 

The histogenesis of carcinosarcomas remains a matter of controversy [11–17]. Some investigators suggested that these tumors develop as a result of undifferentiated, totipotential neoplastic cells that undergo multiple pathways of terminal differentiation into either mesenchymal or epithelial elements. This theory is supported by the presence of epithelial markers (cytokeratin or EMA) in mesenchymal areas and the presence of ultrastructural features (desmosomes or tonofilaments) of epithelial differentiation in sarcomatoid elements [11]. Others believe that in cases where different components share no common features on immunohistochemical and electron microscopic examinations, carcinosarcomas might be the result of true “collision” tumors, where both malignant epithelial and mesenchymal components arise independently from each other [15]. Several investigators evaluated clonality in both malignant epithelial and mesenchymal elements using genetics or molecular techniques. The tumor cells from both tumor components showed monoclonality and clonal identity in all cases studied, suggesting a monoclonal origin [12, 13, 16].

In contrast to well-described histopathological findings, the demographic features and clinical behavior of these tumors remain ill-defined in this disease, and the issues of the prognosis, treatment of those tumors have rarely been specifically addressed. In this population-based study, a comprehensive analysis of patients with carcinosarcoma of the urinary bladder identified in the SEER Program database was performed.

## 2. Methods

### 2.1. Data Source

Surveillance, Epidemiology, and End Results (SEER) databases included patient records from multiple sites across the United States. The database was designed to reflect overall characteristics of the United States, including the variety of racial/ethnic groups, and geographical locations. SEER 9, 13, and 17 registries cover approximately 9.5%, 13.8%, and 26.2% of the total U.S. population, respectively [18]. Data for this study were obtained from SEER*Stat public-use data files, available on the internet at the National Cancer Institute web site.

### 2.2. Study Population

The cases of carcinosarcoma were extracted from the SEER on the basis of anatomic site (ICD-O-2 codes C67.0–C67.9) and histologic type (ICD-O code 8980 and 8981) for those patients first diagnosed and/or treated between January 1973 and December 2004. 198,317 adult patients with bladder neoplasms were identified in the SEER 17 registries, including 221 patients with carcinosarcoma of the urinary bladder. 

The SEER system collects data regarding extent of disease at diagnosis, and classifies patients as having local, regional (extension into adjacent tissues or nodal involvement), or distant disease. It incorporates the World Health Organization's standard grading system used with four separate categories (well, moderately well, poorly differentiated, and undifferentiated) [18].

### 2.3. Statistical Analysis

SEER*Stat 6.2.4 (Surveillance Research Program, National Cancer Institute) was used for incidence analysis [18]. Age-adjusted incidence rates and their 95% CIs were calculated for carcinosarcoma of the urinary bladder for all patients, for men and women separately, and for each of the 3 broad categories of race (whites, blacks, and other). For calculation of the age-adjusted incidence rates, the US general population for the year 2000 was used as a standard population. 

Discrete data are reported as frequencies and compared by chi-square and Fisher's exact tests as appropriate. Continuous data are reported as mean ± SD and compared by student's *t*-test. Cases identified at the time of autopsy or by death certificate only or with more than one primary were excluded from survival analyses. Survival duration was measured by the Kaplan-Meier method [19] and compared by the log rank test. The statistical independence between prognostic variables was evaluated by multivariate analysis by the Cox proportional hazard model [20]. All other statistical calculations were performed by SPSS 12.0 (Apache Software Foundation 2000). Comparative differences were considered statistically significant when the *P* value was <.05.

## 3. Results

### 3.1. Frequency and Incidence

Between 1973 and 2004, a total of 198,317 patients with bladder neoplasms were identified in the SEER 17 registries, including 221 (0.11%) patients with histologically confirmed carcinosarcoma of the urinary bladder. 

Using linked population files, the incidence of carcinosarcoma of the urinary bladder was calculated as a rate per 100,000 per year, age adjusted to year 2000 U.S. standard population. An age-adjusted incidence of 0.02 per 100,000 was observed (2 per 10,000,000 persons per year). Detailed incidence data by gender and race are included in Table 1.

### 3.2. Patient and Tumor Characteristics

The median age at diagnosis was 75 years, with a range of 41 to 96 years. The male to female ratio was 1.9 : 1. The majority of patients (89.1%) were white, while African American accounted for 6.8%. Other ethnicities accounted for 4.1%. Details of patient and tumor characteristics of study cohort are included in Table 2.

The most common location of carcinosarcomas was the lateral wall of the bladder, with the dome, trigon, anterior, and posterior walls being less common. Of the 127 patients whose histology grade was available, 125 (98.4%) had poorly or undifferentiated histology. Of 204 patients with known SEER stage, 56 (27.5%) patients had localized stage; 114 (55.9%) had regional stage; and 34 (16.7%) had distant stage. A relatively large percentage (39.8%) of patients had two or more primary tumors.

### 3.3. Treatment

Most of patients (95.9%) were treated with cancer directed surgery (CDS), 35 (15.8%) of patients received radiation therapy combined with cancer-directed surgery. One patient received radiation before and after surgery; 34 patients received radiation after surgery. Among the patients who received cancer directed surgery, 119 (53.9%) patients underwent transurethral resection for bladder tumor only and 79 (35.7%) patients underwent partial or radical cystectomy. In 3.2% of cases, no surgical or radiation therapy was given after the diagnosis was established (Table 2).

### 3.4. Survival

The median duration of followup of the entire cohort was 9 months (range 0–227 months); the median duration of followup for censored patients was 162 months. 182 of 221 (82.4%) patients died during the followup period.

**Figure 1:**
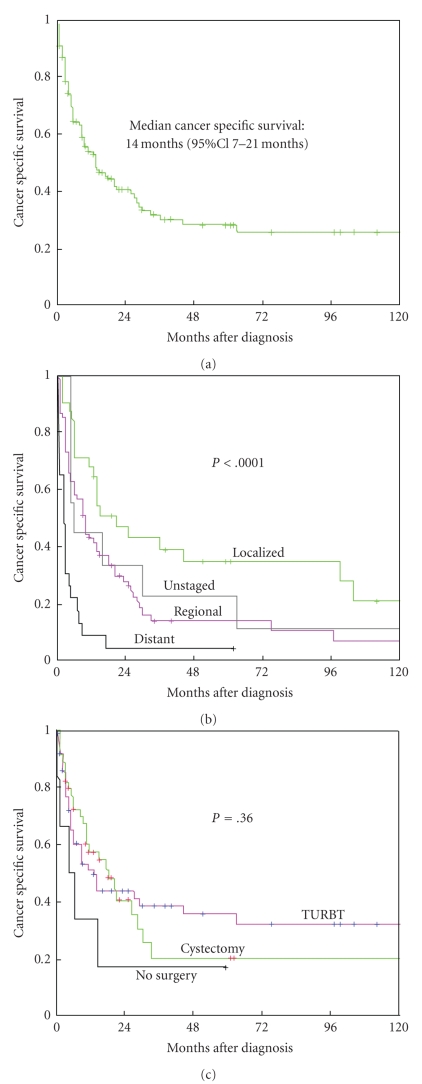
(a) Cancer-specific survival rate of patients with carcinosarcoma of the bladder. (b) Cancer-specific survival rate according to SEER stage. (c) Cancer-specific survival rate according to status of cystectomy (TURBT: transurethral resection for bladder tumor).

For bladder cancer specific survival analyses, the cases that were identified at autopsy or on the basis of death certificates only as well as those patients with more than one primary tumor were excluded. A total of 132 patients were included in bladder cancer specific survival analysis. The median cancer specific survival was 14 months (95% CI 7–21 months) (Figure 1(a)).

Table 3 presents the 1-, 5-, and 10-year cancer-specific survival rate according to patients and tumor characteristics. There were significant differences of cancer-specific survival among SEER tumor stage groups (Figure 1(b)). However, no significant difference of cancer-specific survival was seen for those who underwent cystectomy or transurethral resection of bladder tumor (Figure 1(c)).

In a multivariate survival analyses by Cox proportional hazard modeling, only tumor stage was identified as an independent factor associated with cancer-specific survival. Compared to patients with localized disease, patients with regional and distant disease had a 2.2-and 8.9-fold increased risk of dying from bladder cancer, respectively (Table 4).

## 4. Discussion

Because of the rarity of carcinosarcoma of the urinary bladder, previously published information has been based on case series and anecdotal experiences. The clinical significance and biologic behavior of this subtype of primary bladder cancer needs to be further characterized by performing more extensive studies with long-term followup. This study, took the advantage of the vast amount of data collected by the SEER Program to examine the largest series of carcinosarcoma of the urinary bladder reported to date, and represents the first population-based study of carcinosarcoma of the urinary bladder in published literature. 

Most patients with carcinosarcoma of the urinary bladder in this study had high-histological grade and advanced stage disease at the time of presentation: 148 of 204 (72.5%) patients with known stage were classified as having regional or distant stage; 125 of 127 (98.4%) patients with known histology grade, had poorly or undifferentiated histology (Table 2). These findings were consistent with prior reports that carcinosarcoma of the bladder is a highly aggressive subtype of bladder cancer [2–10].

Approximately, 40% of the bladder carcinosarcoma patients in this population were affected by multiple primary tumors. The previously underrecognized high incidence of multiple primaries in patients with carcinosarcoma of the urinary bladder suggests a common underlying mechanism or pathway of carcinogenesis. Although the potential nonrandom association and causal relationship between carcinosarcoma and other neoplasms remains unknown, field cancerization, cancer stem cell, or patient screening effect may account for the relatively frequent association of multiple primaries in patients with urogenital tumors [21–23].

Owing to the rareness of carcinosarcoma, and in the absence of randomized controlled trials, there is no standard treatment for this disease. In contrast to non-muscle-invasive transitional cell carcinoma of the bladder, non muscle-invasive carcinosarcoma of the urinary bladder usually involves the lamina propria. In addition to the carcinomatous degeneration of the mucosa, sarcomatous degeneration of the underlying submucosal stroma is also present [24]. TURBT (transurethral resection for bladder tumor) or partial cystectomy, carries the risk of incomplete tumor removal. Therefore, radical cystectomy appears to be the treatment of choice for both superficial and deeply invasive disease [25]. For muscle invasive disease, some authors advocate radical treatment (i.e., cystectomy) whenever possible. Even though, local recurrence and/or metastasis rates were very high after radical surgery [24]. 

Consistent with single institution studies, the cancer specific survival of this cohort of carcinosarcoma of the urinary bladder was poor. In our study, the 1-, 5- and 10-year survival rate of carcinosarcoma of the urinary bladder were 53.9%, 28.4%, and 25.8% (Figure 1(a)), which is much lower than the 5-year overall survival rate of bladder cancer as whole [25]. In a multivariate survival analyses by Cox proportional hazard modeling, tumor stage was the only factor independently associated with cancer-specific survival. Similarly, a previous report showed a mortality rate of 80% at a mean followup of 14 months with pathologic stage being the best single predictive factor of survival [9]. Tumor stage also has been shown to be a strong predictor for survival in patients with other subtypes of bladder cancer such as urothelial carcinoma, squamous cell carcinoma, adenocarcinoma, and small cell carcinoma [24, 26]. The findings of this study, along with others [9], underscore the importance of early detection and diagnosis in this disease.

The prognosis of this tumor remains poor, even in patients with resectable disease. The overall 5-year cancer-specific survival rate after cystectomy in our study population was only 20.3%, suggesting a high risk of early dissemination. Recently, cystoprostatectomy with lymphadenectomy plus various combinations of neoadjuvant or adjuvant chemotherapy and/or radiotherapy has been advocated by some authors [24, 26–29]. However, the outcomes have been variable and inconsistent. While some authors reported promising results with ovarian or sarcoma-type chemotherapy regimens [30], others reported a poor outcomes regardless of the type of treatment [9]. Although the combination of gemcitabine and cisplatin has been an effective and well-tolerated chemotherapy regimen for the treatment of advanced urothelial carcinoma [31], there are only a few case reports regarding its use in carcinosarcoma of the urinary bladder [29, 32]. Multi-institution clinical trials are needed to establish a better therapeutic protocol for this rare but aggressive cancer. 

Strengths of this study include the population-based design with a large sample size and the inclusion of a wide range of age and racial groups in the analysis. Large sample size is of particular importance for analysis of rare tumors such as carcinosarcoma of the urinary bladder, where it is nearly impossible for a single institution to collect enough cases to make meaningful predictions regarding important prognostic factors and treatment recommendations.

Limitations of the study include of the lack central review of pathology reports. In addition, information regarding receipt of chemotherapy or patients' comorbidities is not available in SEER database, all of which may influence survival in cancer patients. However, the use of specific survival rather than overall survival in our study has modified the limitation to some degree. Finally, sample size in our study may still not be enough to fully describe the factors that affect the incidence, treatment choice, and survival of this rare tumor.

## 5. Conclusion

In summary, carcinosarcoma of the urinary bladder is a highly malignant neoplasm, occurring predominantly in elder males with an advanced stage at presentation and was rapidly lethal. A better understanding of the natural history of the disease and prognostic factors as provided herein are necessary to allow physicians and patients to accurately assess the risks and potential benefits of treatment. Future advances in the molecular biology of this disease may lead to development of novel treatment strategies for this relatively rare but complex disease.

## Figures and Tables

**Table 1:** Age adjusted incidence rate of sarcomatoid carcinoma (carcinosarcoma) of the urinary bladder per 100,000 populations.

Age-adjusted incidence rate*95% CI		

Overall	0.02	0.02-0.03
Gender		
Male	0.03	0.03-0.04
Female	0.01	0.01-0.02

Race		
White	0.02	0.02-0.03
Black	0.02	0.01–0.04
Others	0.01	0.00–0.03

*Rates are per 100,000 population (95% confidence interval) age-adjusted to year 2000 U.S. Standard population.

**Table 2:** Characteristics of 221 patients with sarcomatoid carcinoma (carcinosarcoma) of the urinary bladder diagnosed between January 1973 and December 2004.

Characteristics	*N *(%)
Age Mean ± SD	75 ± 11
Gender	
Male	144 (65.2)
Female	77 (34.8)

Race	
Black	15 (6.8)
White	197 (89.1)
Other	9 (4.1)

Married	
Yes	128 (58)
No	90 (40.7)
Unknown	3 (1.3)

Location	
Trigone of bladder	12 (5.4)
Dome of bladder	17 (7.7)
Lateral wall of bladder	35 (15.8)
Anterior wall of bladder	8 (3.6)
Posterior wall of bladder	20 (9.0)
Bladder neck	7 (3.2)
Ureteric orifice	7 (3.2)
Overlapping lesion of bladder	42 (19.0)
Unknown	73 (33.0)

Grade	
Moderately differentiated	2 (0.9)
Poorly differentiated	61 (27.6)
Undifferentiated	64 (28.95)
Unknown	94 (42.5)

SEER stage	
Localized	56 (25.3)
Regional	114 (51.6)
Distant	34 (15.4)
Unknown	17 (7.7)

Year of diagnosis	
1973–1984	18 (8.1)
1985–1994	65 (29.4)
1995–2004	138 (62.5)

Cancer directed Surgery	
Cystectomy	79 (35.8)
TURP	119 (53.8)
Unspecific surgery	14 (6.3)
None	9 (4.1)

Radiation	
Before and after surgery	1 (0.5)
After surgery	34 (15.3)
Radiation only	2 (1.0)
Unknown	3 (1.3)
None	181 (81.9)

SEER = Surveillance, Epidemiology, and End Results program TURBT = transurethral resection for bladder tumor.

**Table 3:** Median, 1-, 5-, and 10-year cancer specific survival of patients with sarcomatoid carcinoma (carcinosarcoma) of the urinary bladder according to demographic and clinical characteristics.

Characteristics		Median survival	P-value	Survival rate (%)		
		Months (95% CI)		1-Year	5-Year	10-Year

All		14 (7, 21)		53.9	28.4	25.8

Tumor stage	Localized	21 (11, 31)	<.001	67.8	34.8	29.0
	Regional	10 (7, 13)		43.2	14.1	7.0
	Distant	2 (1, 3)		8.7	4.4	n/a
	Unstaged	6 (3, 9)		44.4	11.1	11.1

CDS^a^	No surgery	4 (0, 10)	0.36	33.3	16.7	n/a
	TURBT^b^	13 (7,19)		51.2	35.6	32.1
	Cystectomy	18 (7, 29)		57.2	20.3	20.3

^a^CDS: Cancer directed Surgery

^b^TURBT: Transurethral resection for bladder tumor.

**Table 4:** COX proportional multivariate analysis of factors associated with sarcomatoid carcinoma (carcinosarcoma) of the urinary bladder disease specific mortality.

Characteristics	Group	Hazard ratio	95% CI	*P* value
Age	Continuous	1.01	0.99–1.03	.45
Gender	Male	1.00		
	Female	1.26	0.75–2.11	.39

Ethnicity	White	1.00		
	Others	1.28	0.58–2.83	.55

SEER Stage	Localized	1.00		
	Regional	2.17	1.08–4.38	.03
	Distant	8.86	4.03–19.51	<.0001
	Unknown	1.72	0.64–4.62	.28

Married	Yes	1.00		
	No	1.30	0.77–2.20	.33

Diagnose year	1973–1984	1.00		
	1985–1994	0.80	0.32–2.05	.65
	1995–2004	1.15	0.47–2.82	.76

Cystectomy	No	1.00		
	Yes	0.80	0.43–1.50	.49

Radiation	No	1.00		
	Yes	1.15	0.64–2.09	.64

Combination*	No	1.00		
	Yes	0.38	0.08–1.85	.23

HR = Hazard ratio; CI = Confidence interval

*Combination = combination therapy of cystectomy and radiation.
